# Correction: Single nucleus sequencing reveals evidence of inter-nucleus recombination in arbuscular mycorrhizal fungi

**DOI:** 10.7554/eLife.46860

**Published:** 2019-03-21

**Authors:** Eric CH Chen, Stephanie Mathieu, Kinga Sedzielewska-Toro, Max Peart, Adrian Pelin, Steve Ndikumana, Jeanne Ropars, Steven Dreissig, Jorg Fuchs, Andreas Brachmann, Nicolas Corradi

Chen ECH, Mathieu S, Hoffrichter A, Sedzielewska-Toro K, Peart M, Pelin A, Ndikumana S, Ropars J, Dreissig S, Fuchs J, Brachmann A, Corradi N. 2018. Single nucleus sequencing reveals evidence of inter-nucleus recombination in arbuscular mycorrhizal fungi. *eLife* **7**:e39813. doi: 10.7554/eLife.39813.

Published 5, December 2018

Recently a reader contacted us about potential inconsistencies between the data in Supplementary File 6 and Figure 2. Figure 2 is directly built from data in Supplementary File 6. After checking the data and the scripts used to generate Figure 2, we found that an earlier version of Figure 2 was mistakenly uploaded.

The earlier version Figure 2 was based on an analysis of the isolate SL1 that allowed the maximum number of Blast hits for regions surrounding SNPs to be of 1, instead of 2 as written in the method section of our paper. The results of this parameter change produced minor differences that do not alter the overall patterns of nuclear diversity originally observed. In all cases, the figure shows that isolates A4 and A5 harbour a structured nuclear genome organization, while in the isolate SL1 nuclear genetic diversity appears to be more diverse.

As a result, Figures 2 (SL1 portion), Supplementary File 10 (SL1 portion), Supplementary File 11 (SL1 portion), Supplemental File 5 (SL1 portion) and Supplementary File 4 (SL1 portion) have been corrected.

Corrected Figure 2:

**Figure 1:**
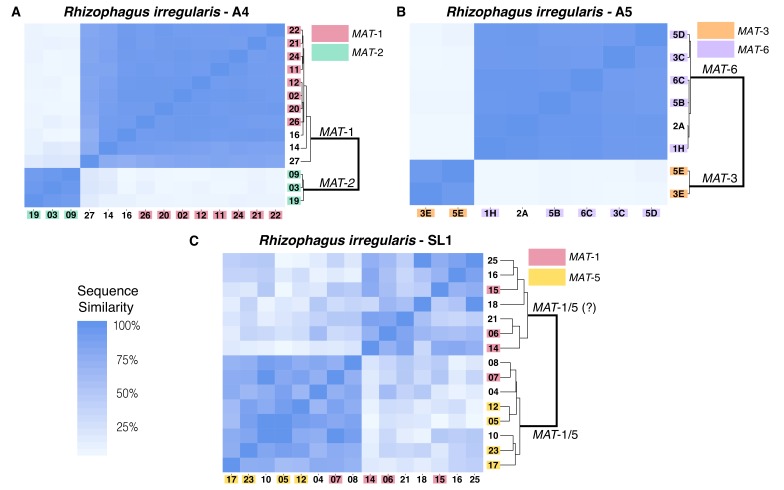


Original Figure 2:

**Figure 2:**
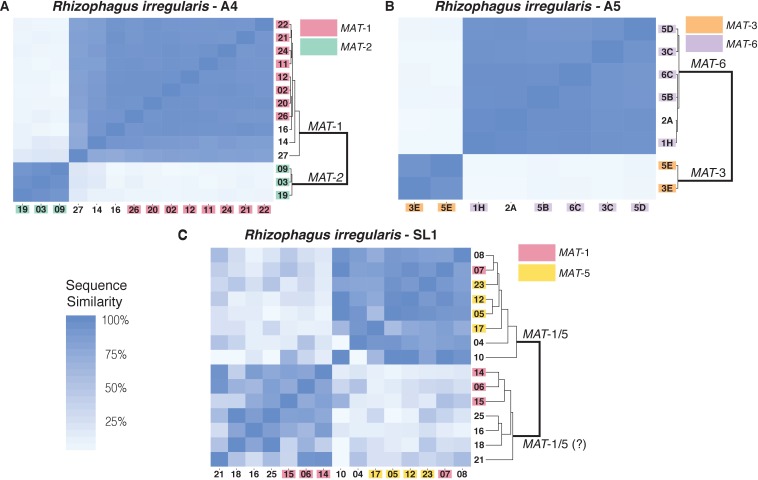


Corrected Supplementary file 10:

**Figure 3:**
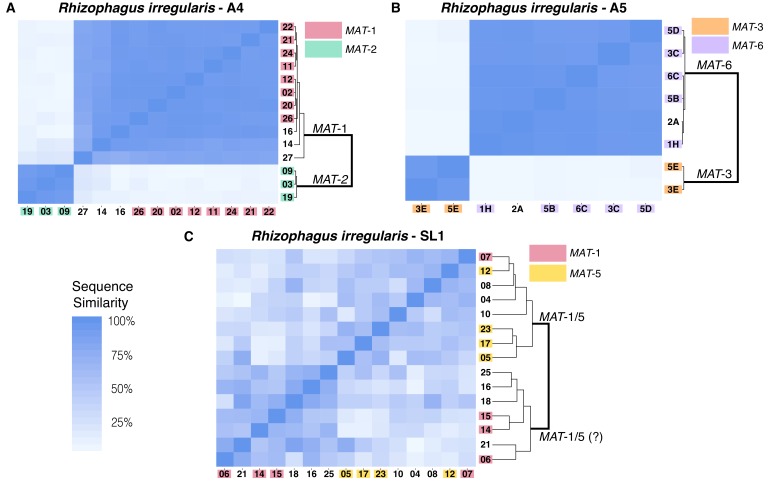


Original Supplementary file 10:

**Figure 4:**
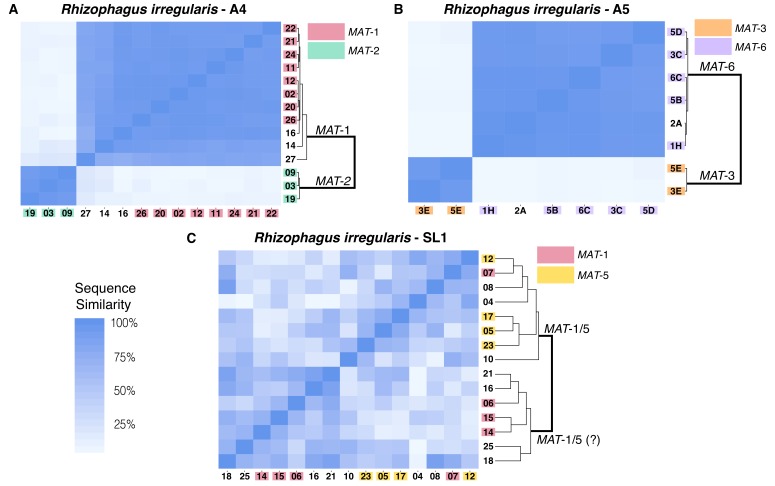


Corrected Supplementary file 11:

**Figure 5:**
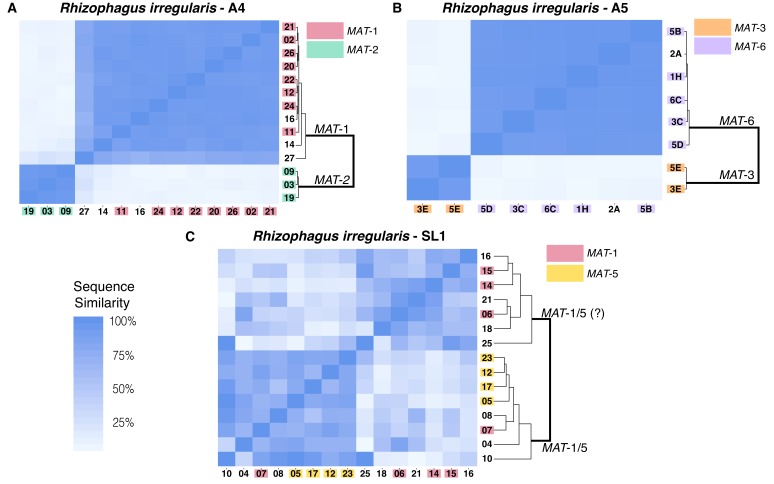


Original Supplementary file 11:

**Figure 6:**
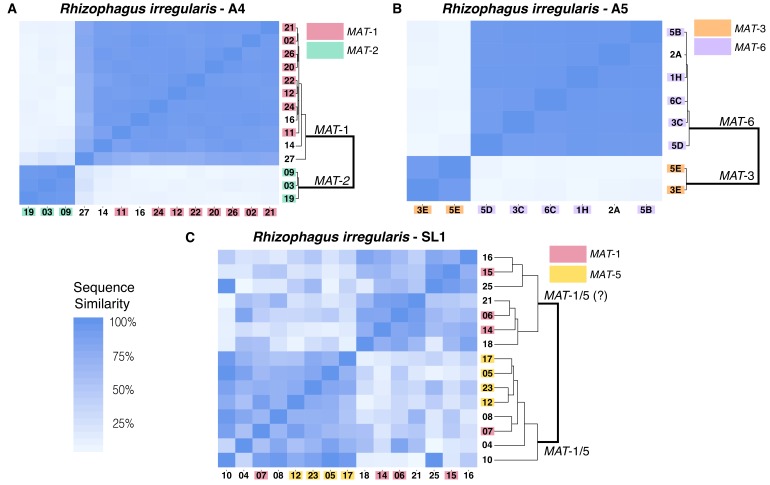


Supplementary file 4 and Supplementary file 5 have also been corrected.

In Supplementary 4 we also identified a correction of number of SNPs in A4 after stringent filtering, from 2363 to 2362. The increase in SNP counts for SL1 with the updated parameter after stringent filter changes the reduction of SNP from 94.20% to 93.81%. The corresponding passage in the article has now been corrected:

Original text: “This conservative approach has likely removed some true positives from our analysis - for example this filtering method resulted in an average 94.2%”

The corrected text now reads as follows:

“This conservative approach has likely removed some true positives from our analysis - for example this filtering method resulted in an average 93.8%”

Our conclusions are unaffected by these changes, and we apologize for this oversight.

The article has been corrected accordingly.

## Additional files

Supplementary file 1. Corrected Supplementary file 4.

Supplementary file 2. Original Supplementary file 4.

Supplementary file 3. Corrected Supplementary file 5.

Supplementary file 4. Original Supplementary file 5.

