# Supplementary material for: Single nucleus sequencing reveals evidence of inter-nucleus recombination in arbuscular mycorrhizal fungi
Source: eLife. 2018 Dec 5;7:e39813. doi: 10.7554/eLife.39813 (PMC6281316; doi:10.7554/eLife.39813)
Supplement: Supplementary file 3. — Nuclei that have poor coverage, such as nuclei 13 from A4, were removed. SNPs are distributed fairly evenly across the scaffold although there are apparent hotspots. This may be due to amplification biases. [file elife-39813-supp3.pdf]

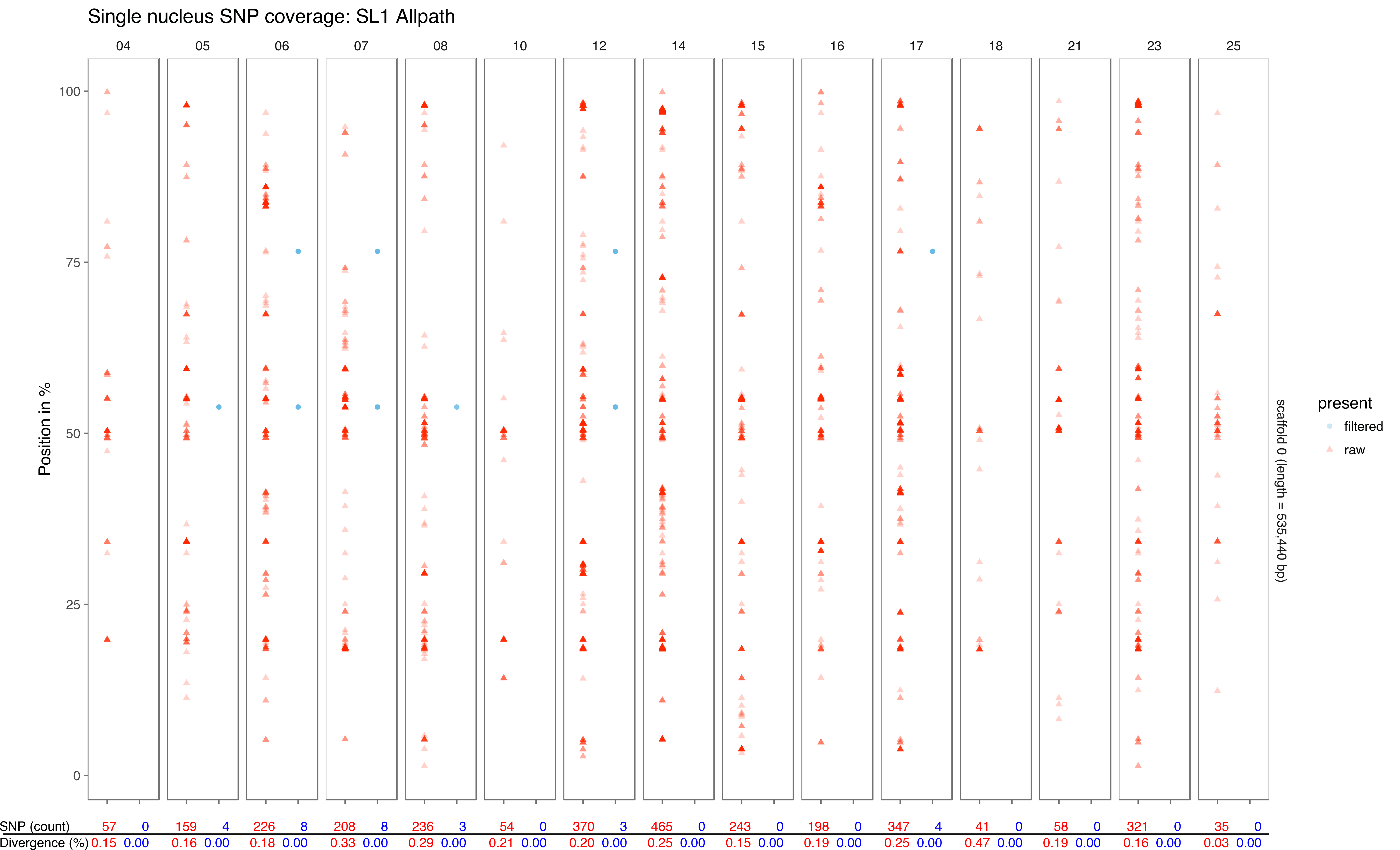
Single nucleus SNP coverage: SL1 Allpath
04 05 06 07 08 10 12 14 15 16 17 18 21 23 25
Position in %
100
75
50
25
0
scaffold 0 (length = 535,440 bp)
present
filtered
raw
SNP (count) 57 0 159 4 226 8 208 8 236 3 54 0 370 3 465 0 243 0 198 0 347 4 41 0 58 0 321 0 35 0
Divergence (%) 0.15 0.00 0.16 0.00 0.18 0.00 0.33 0.00 0.29 0.00 0.21 0.00 0.20 0.00 0.25 0.00 0.15 0.00 0.19 0.00 0.25 0.00 0.47 0.00 0.19 0.00 0.16 0.00 0.03 0.00

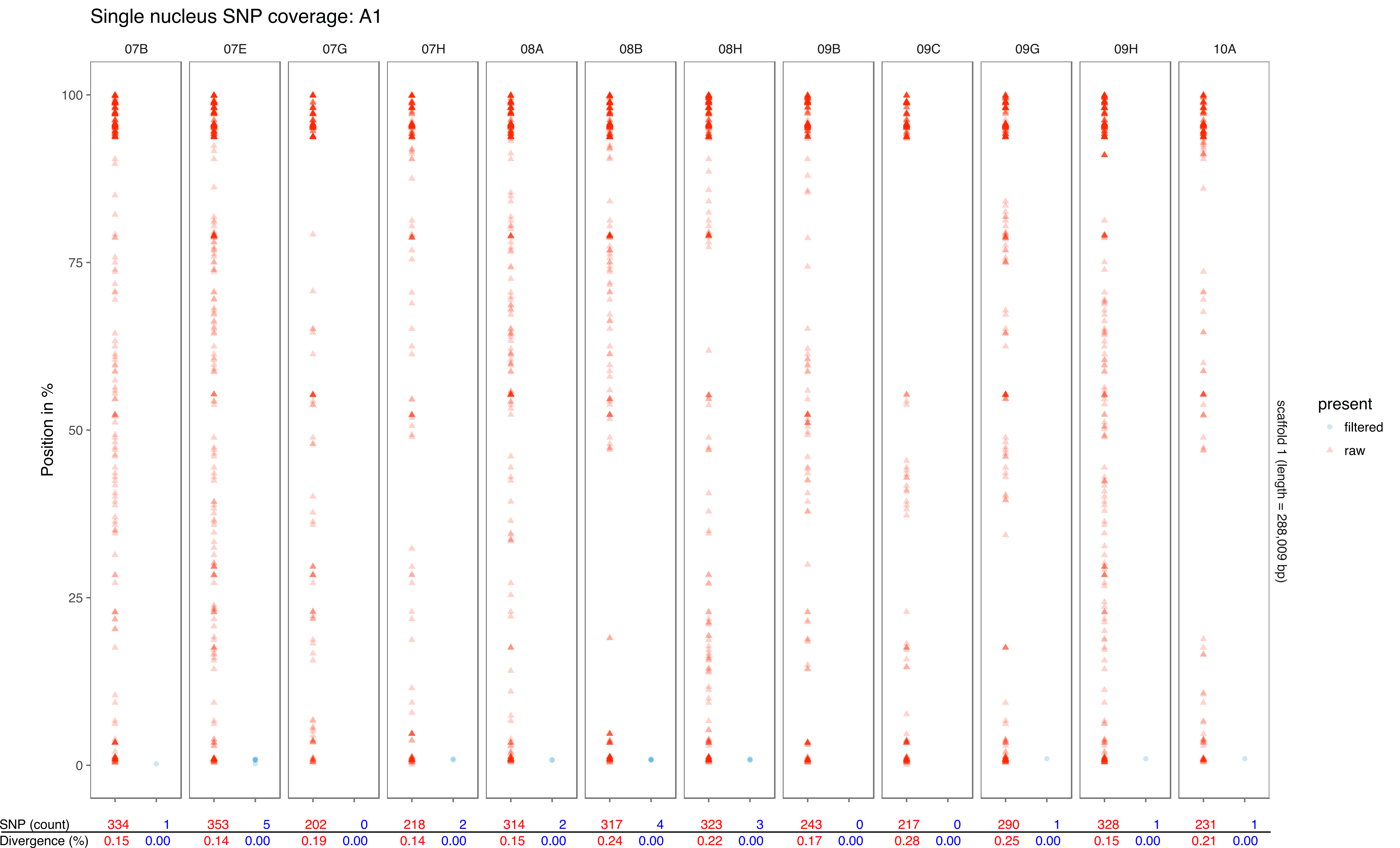
Single nucleus SNP coverage: A1
07B
07E
07G
07H
08A
08B
08H
09B
09C
09G
09H
10A
Position in %
100
75
50
25
0
scaffold 1 (length = 288,009 bp)
present
filtered
raw
SNP (count) 334 1 353 5 202 0 218 2 314 2 317 4 323 3 243 0 217 0 290 1 328 1 231 1
Divergence (%) 0.15 0.00 0.14 0.00 0.19 0.00 0.14 0.00 0.15 0.00 0.24 0.00 0.22 0.00 0.17 0.00 0.28 0.00 0.25 0.00 0.15 0.00 0.21 0.00

Single nucleus SNP coverage: A4

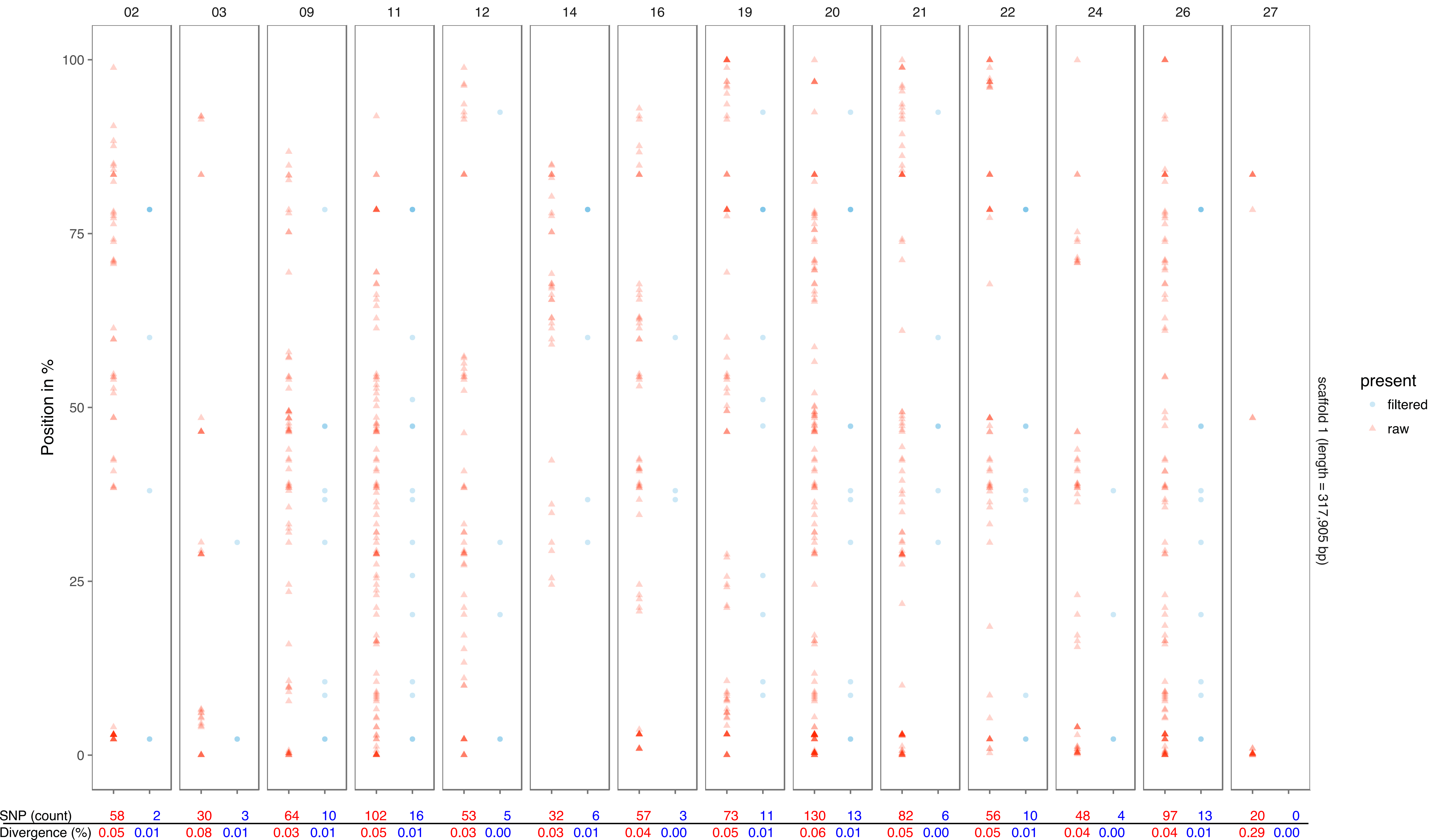

Single nucleus SNP coverage: A5

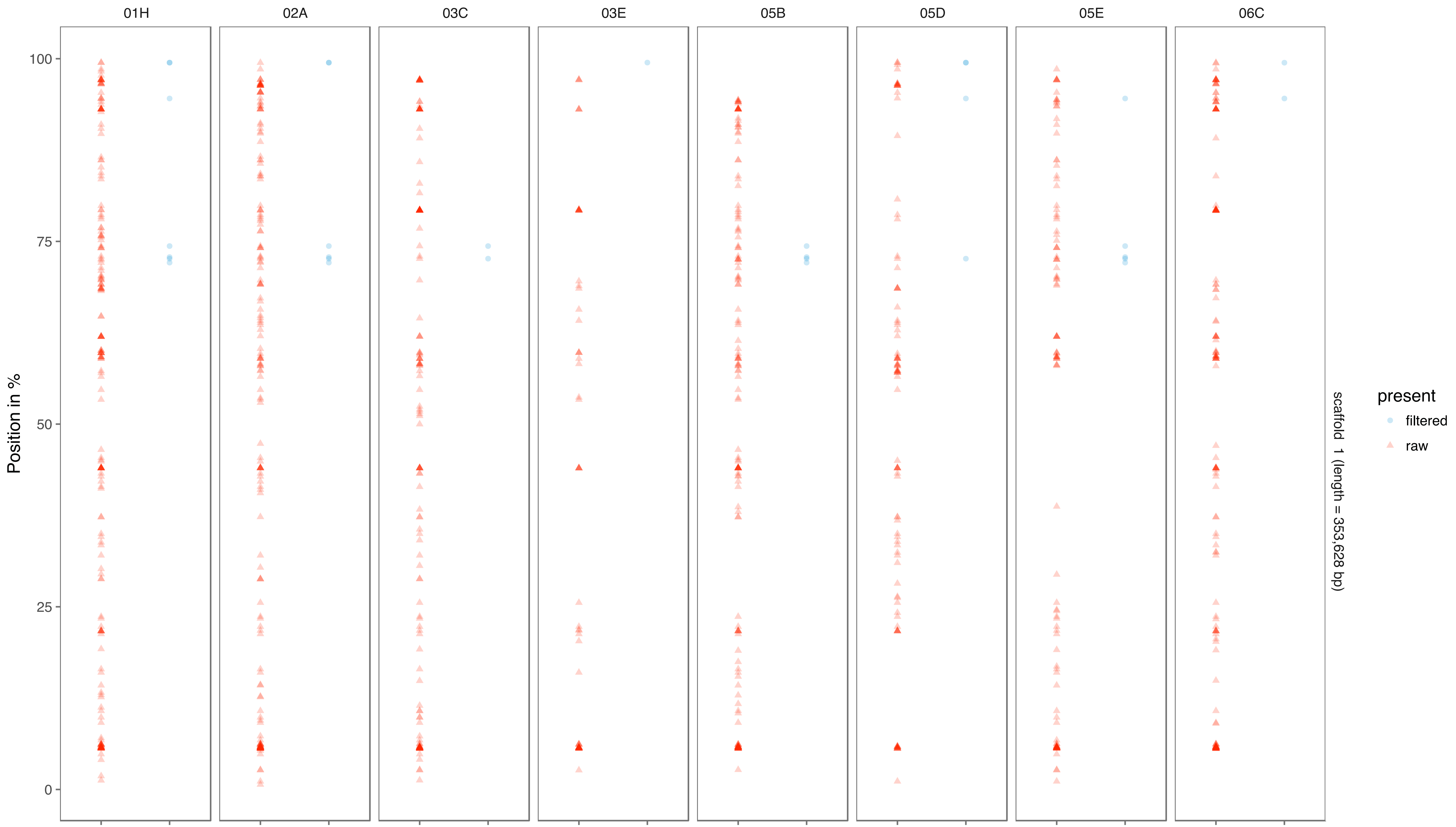

SNP (count)	203	7	186	6	152	2	66	1	156	4	100	4	114	5	156	2
Divergence (%)	0.07	0.00	0.06	0.00	0.07	0.00	0.05	0.00	0.06	0.00	0.05	0.00	0.06	0.00	0.08	0.00

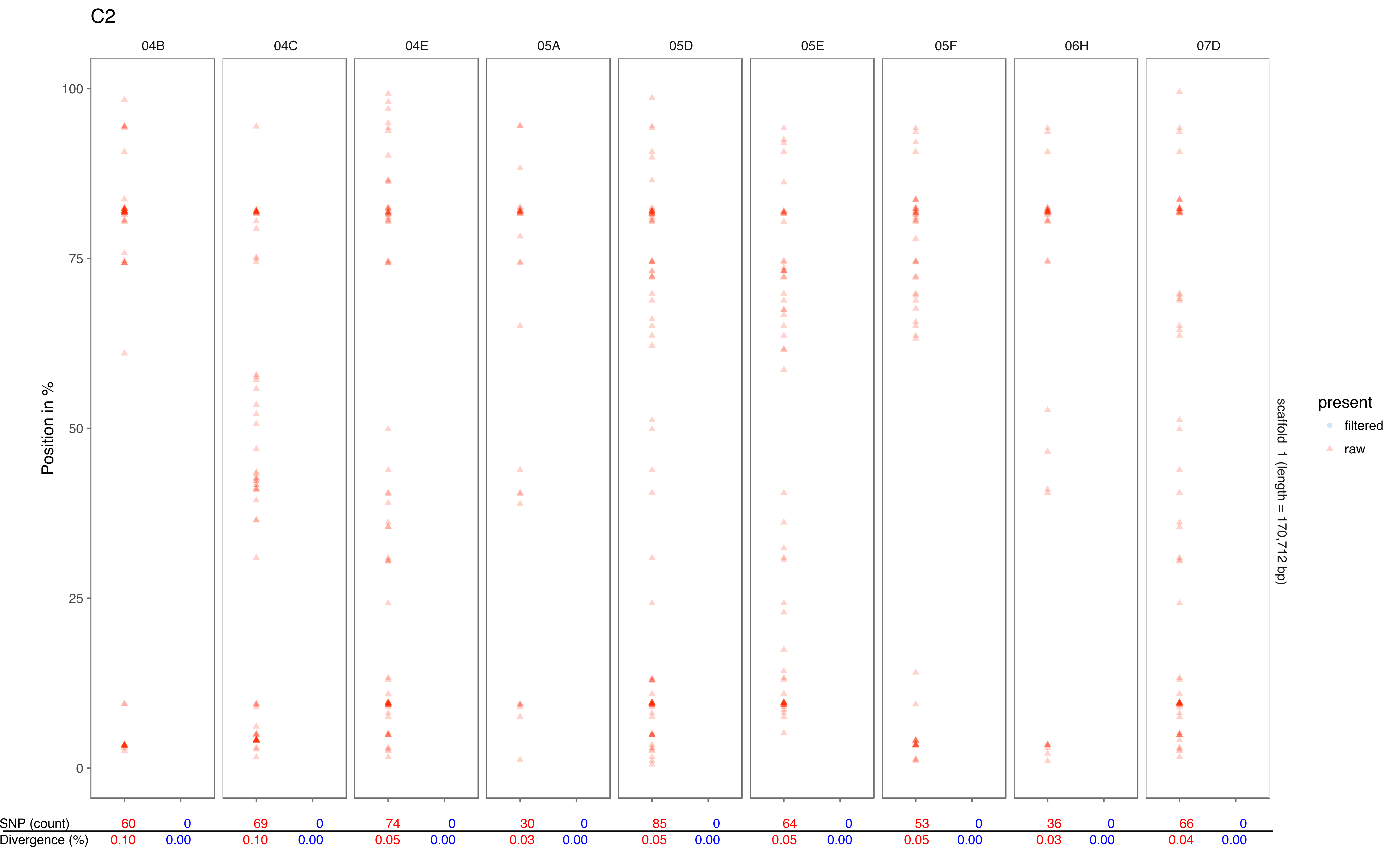

C2
04B
04C
04E
05A
05D
05E
05F
06H
07D
Position in %
100
75
50
25
0
scaffold 1 (length = 170,712 bp)
present
filtered
raw
SNP (count)
60 0 69 0 74 0 30 0 85 0 64 0 53 0 36 0 66 0
Divergence (%)
0.10 0.00 0.10 0.00 0.05 0.00 0.03 0.00 0.05 0.00 0.05 0.00 0.05 0.00 0.03 0.00 0.04 0.00

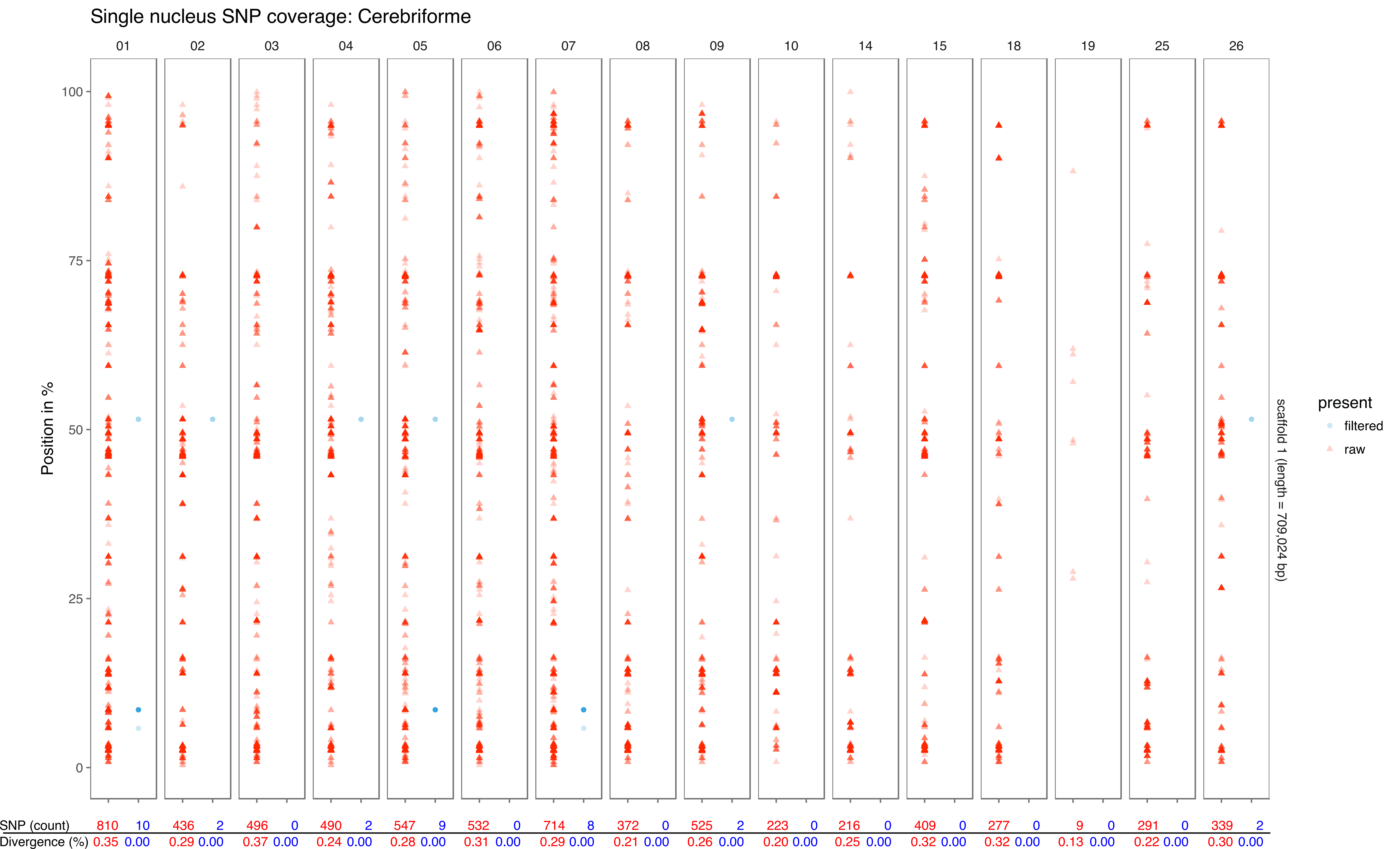
Single nucleus SNP coverage: Cerebriforme
01
02
03
04
05
06
07
08
09
10
14
15
18
19
25
26
100
75
50
25
0
Position in %
scaffold 1 (length = 709,024 bp)
present
filtered
raw
SNP (count) 810 10 436 2 496 0 490 2 547 9 532 0 714 8 372 0 525 2 223 0 216 0 409 0 277 0 9 0 291 0 339 2
Divergence (%) 0.35 0.00 0.29 0.00 0.37 0.00 0.24 0.00 0.28 0.00 0.31 0.00 0.29 0.00 0.21 0.00 0.26 0.00 0.20 0.00 0.25 0.00 0.32 0.00 0.32 0.00 0.13 0.00 0.22 0.00 0.30 0.00

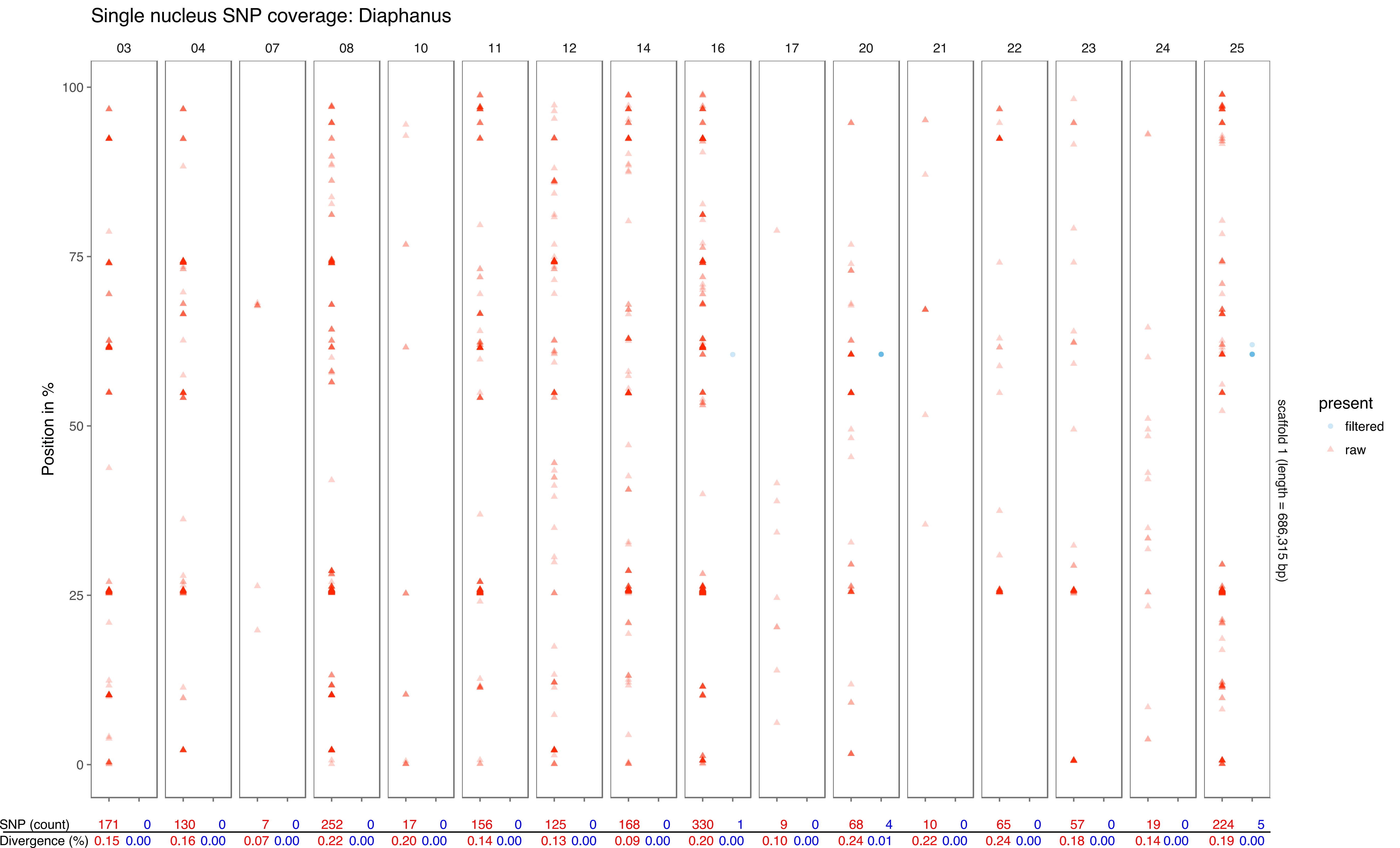
Single nucleus SNP coverage: Diaphanus
03 04 07 08 10 11 12 14 16 17 20 21 22 23 24 25
Position in %
100
75
50
25
0
scaffold 1 (length = 686,315 bp)
present
filtered
raw
SNP (count) 171 0 130 0 7 0 252 0 17 0 156 0 125 0 168 0 330 1 9 0 68 4 10 0 65 0 57 0 19 0 224 5
Divergence (%) 0.15 0.00 0.16 0.00 0.07 0.00 0.22 0.00 0.20 0.00 0.14 0.00 0.13 0.00 0.09 0.00 0.20 0.00 0.10 0.00 0.24 0.01 0.22 0.00 0.24 0.00 0.18 0.00 0.14 0.00 0.19 0.00
