# Supplementary figures and images for: Single nucleus sequencing reveals evidence of inter-nucleus recombination in arbuscular mycorrhizal fungi
Source: eLife. 2018 Dec 5;7:e39813. doi: 10.7554/eLife.39813 (PMC6281316; doi:10.7554/eLife.39813)

**A** *Rhizophagus irregularis* - A4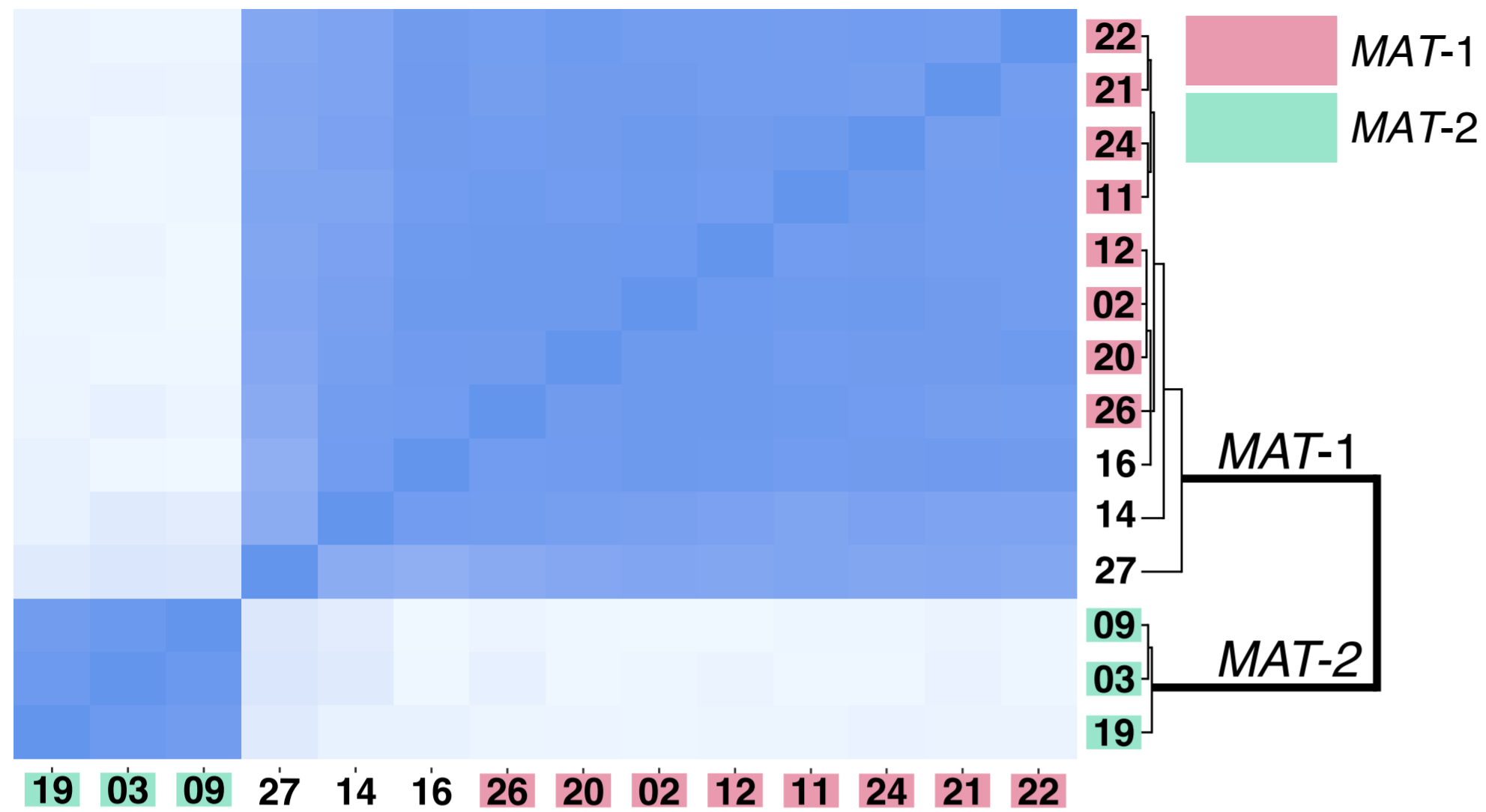**B** *Rhizophagus irregularis* - A5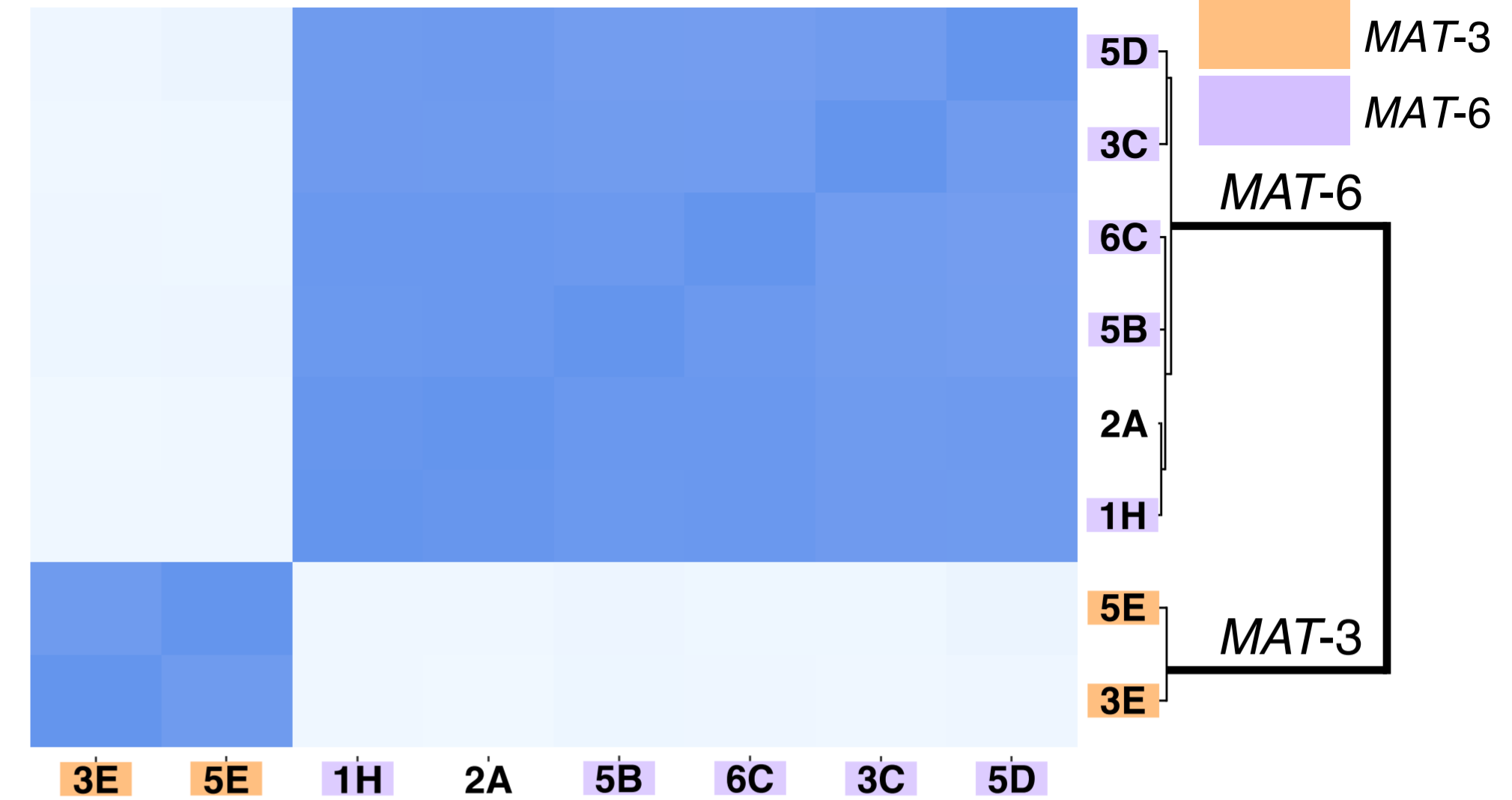**C** *Rhizophagus irregularis* - SL1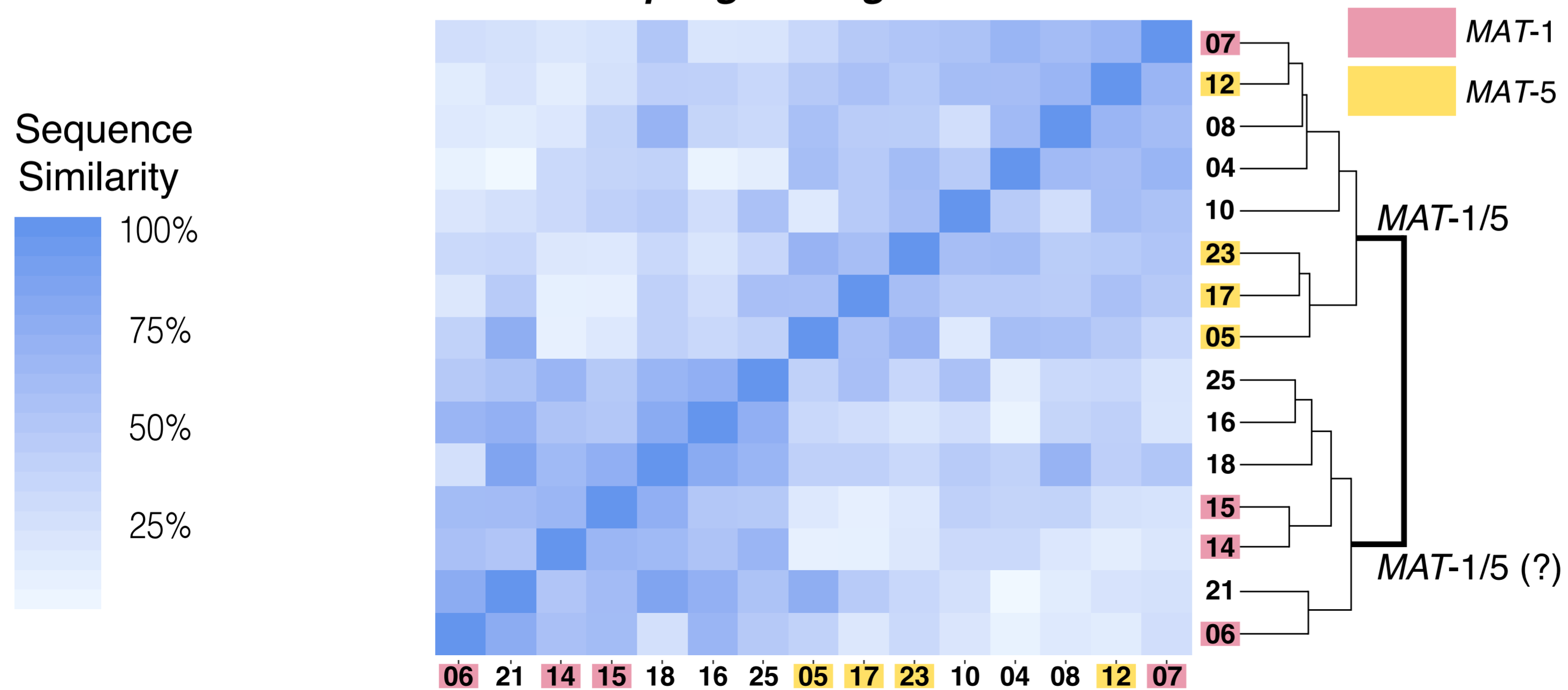

Supplement: Supplementary file 10. — Same as Figure 2 but instead of using SPAdes version of SL1 assembly, the ALLPATH-LG version of SL1 assembly is used. The number ID of the nuclei with a MAT-locus and genotype verified using PCR and Sanger sequencing are shown in coloured boxes. The patterns remain the same, with A4 and A5 having clear segregation of nuclei based on MAT-locus with SL1 showing a much more mosaic pattern. [file elife-39813-supp10.pdf]

**A** *Rhizophagus irregularis* - A4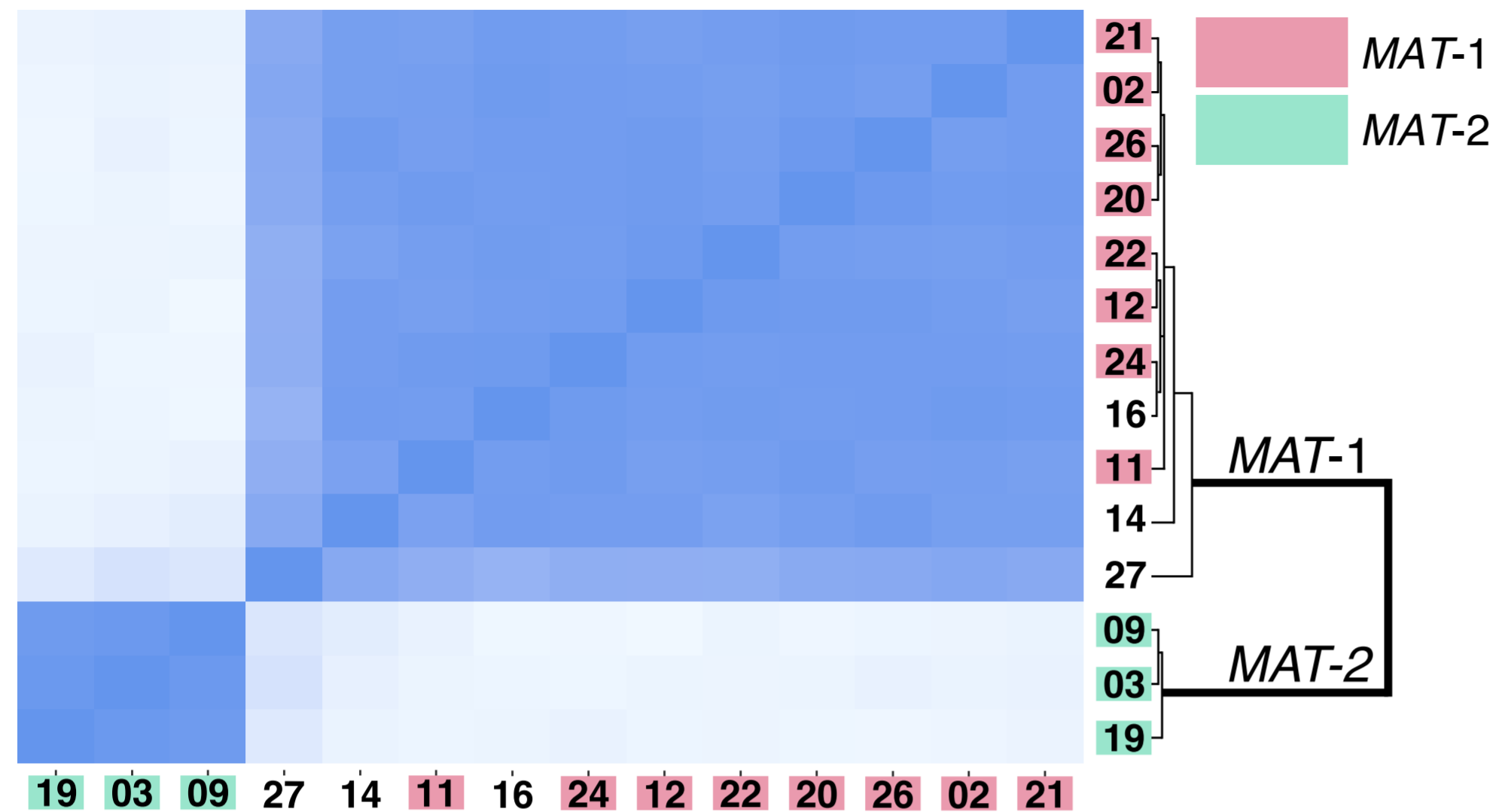**B** *Rhizophagus irregularis* - A5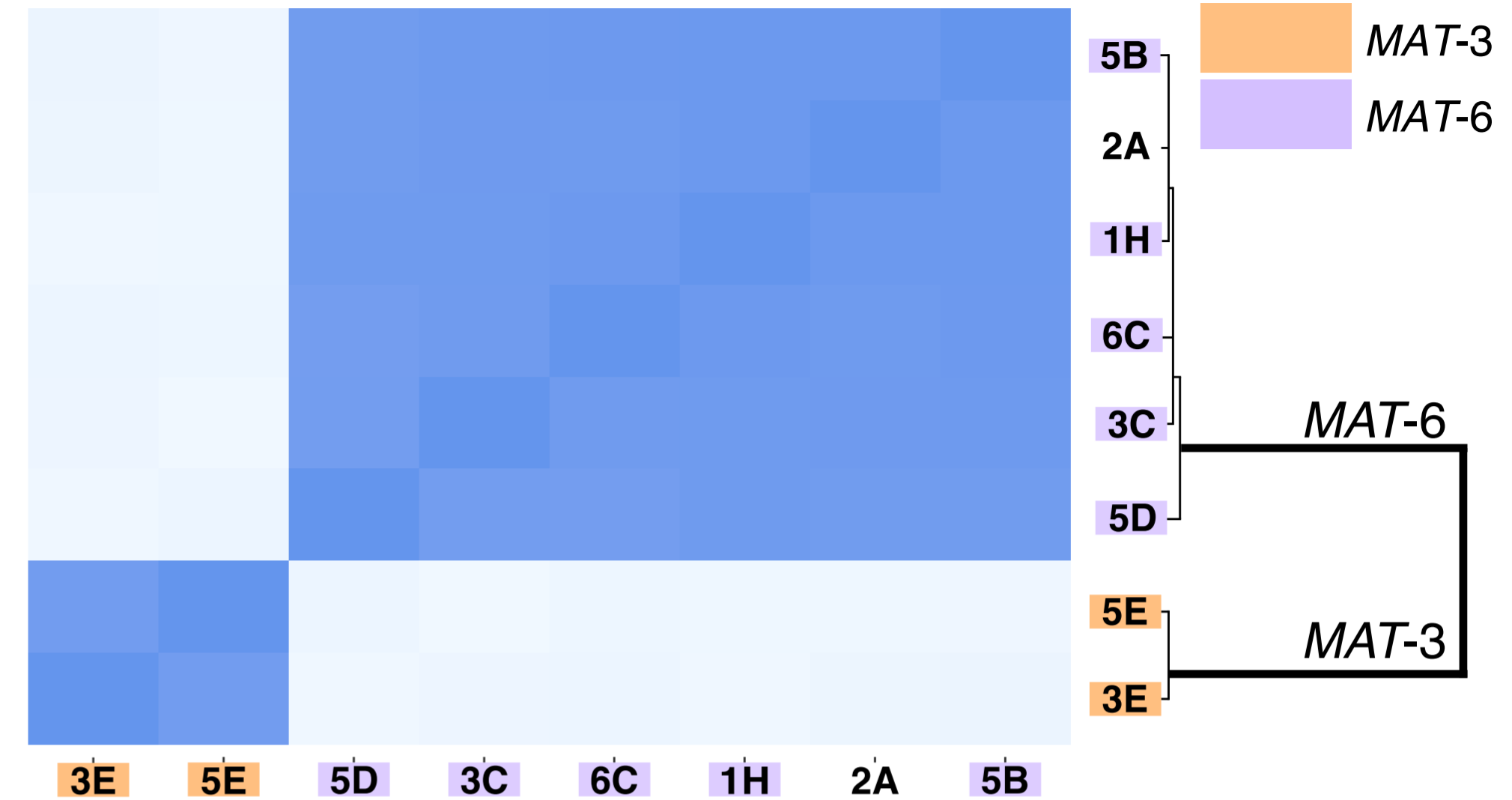**C** *Rhizophagus irregularis* - SL1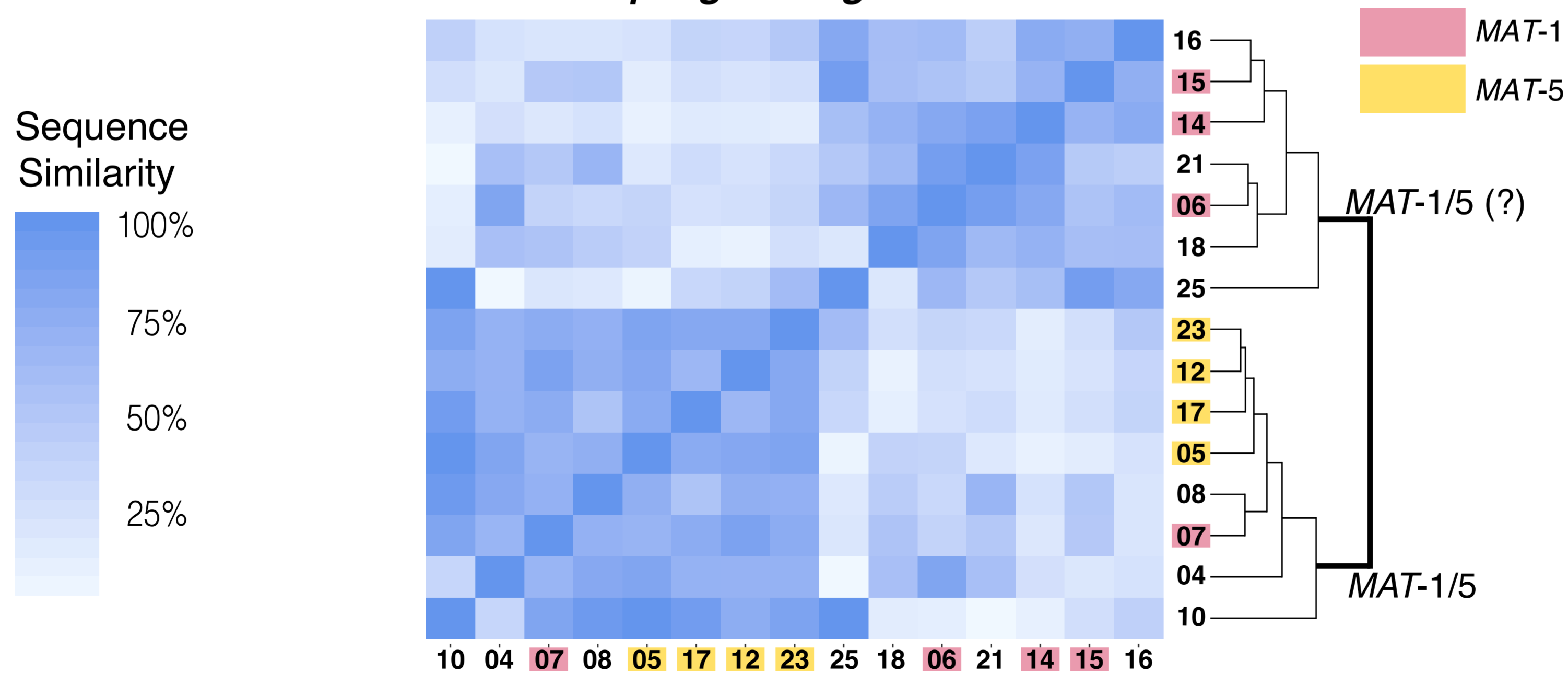

Supplement: Supplementary file 11. — Same as Figure 2 but we used GATK’s HaplotypeCaller instead of freebayes for SNP calling. The number ID of the nuclei with a MAT-locus and genotype that were verified using PCR and Sanger sequencing are shown in coloured boxes. The patterns remain the same, with A4 and A5 having clear segregation of nuclei based on MAT-locus with SL1 showing a much more mosaic pattern. [file elife-39813-supp11.pdf]

**A*****Rhizophagus irregularis* - A4**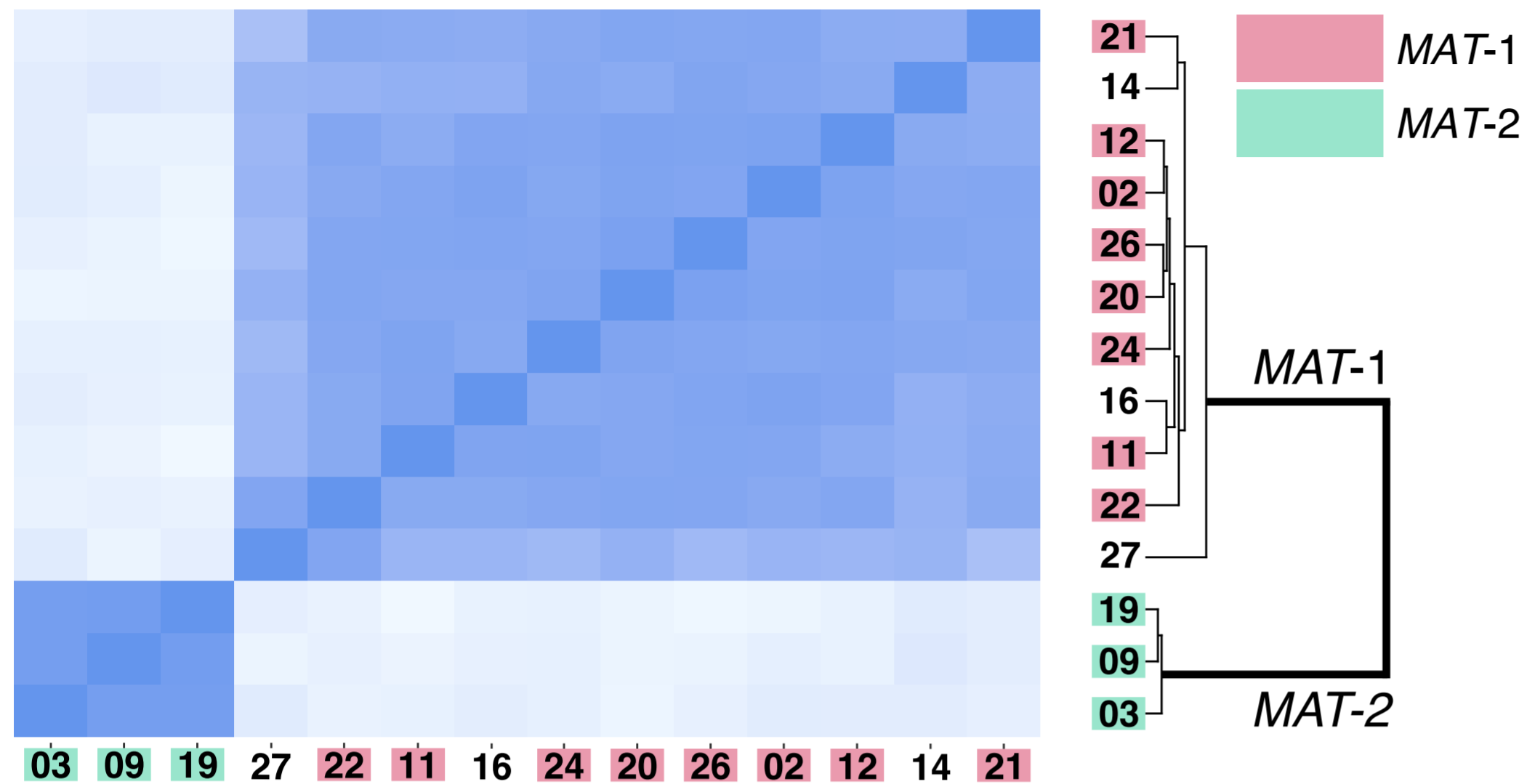**B*****Rhizophagus irregularis* - A5**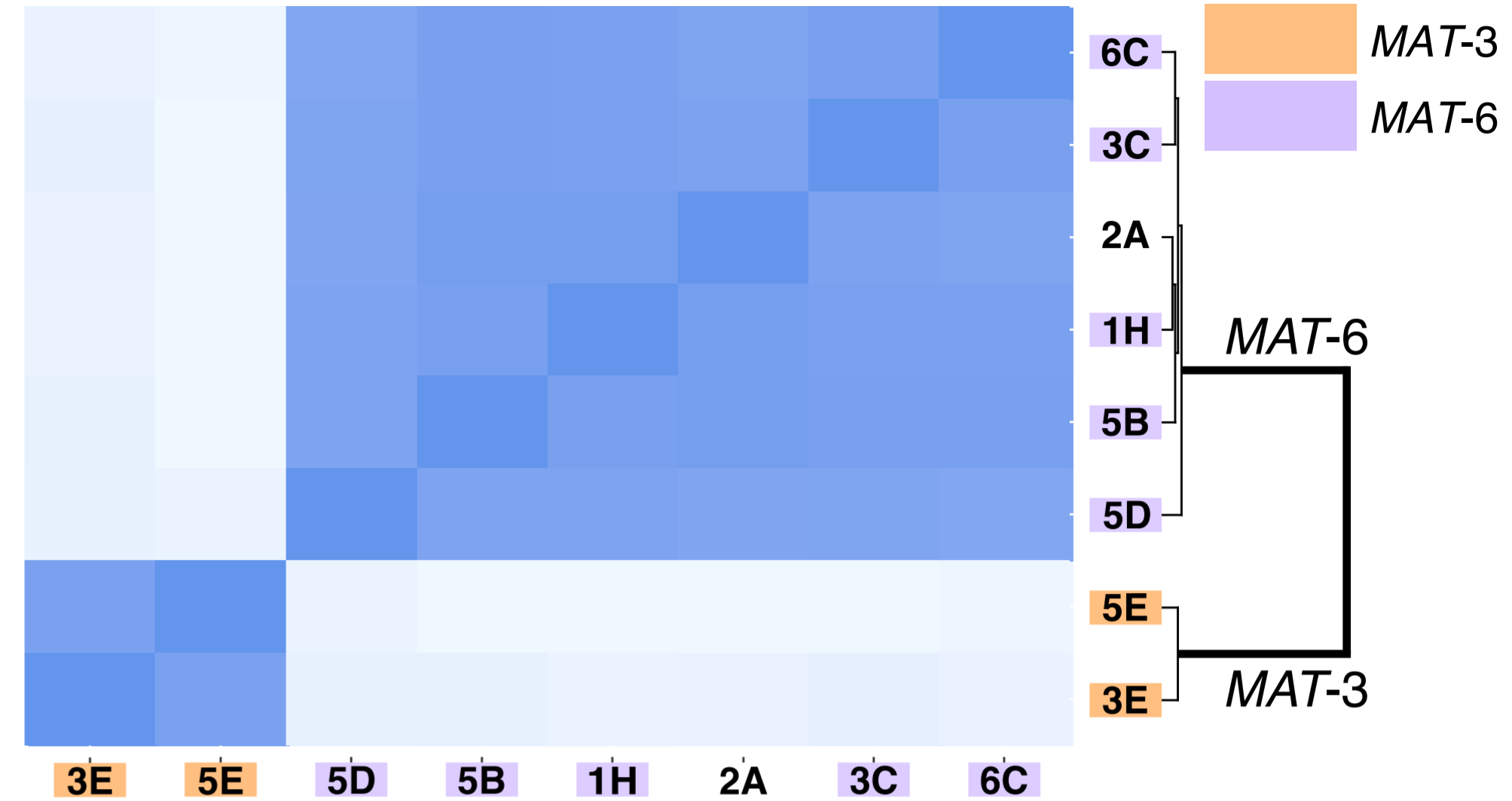**C*****Rhizophagus irregularis* - SL1**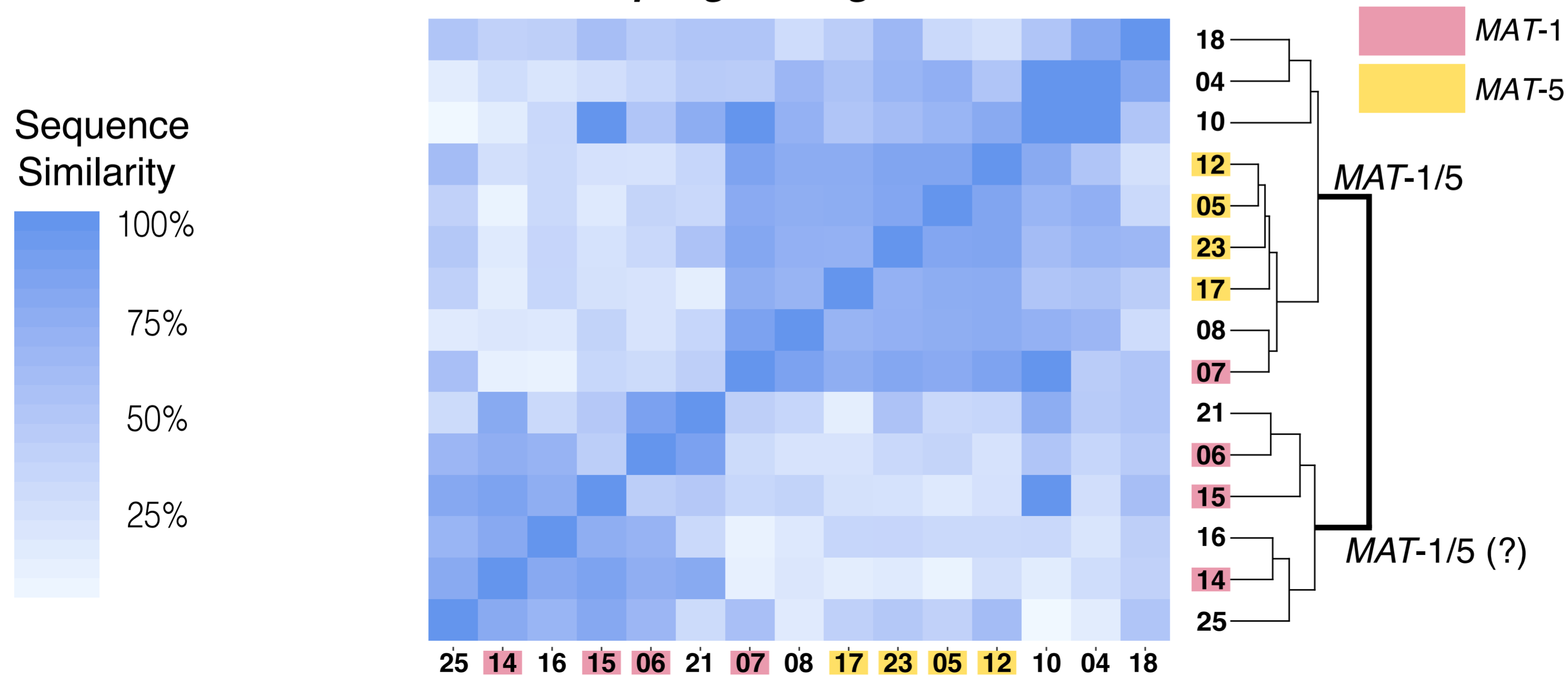

Supplement: Supplementary file 12. — Same as Figure 2 but we used GATK’s Mutect2 instead of freebayes for SNP calling. The number ID of the nuclei with a MAT-locus and genotype that were verified using PCR and Sanger sequencing are shown in coloured boxes. The patterns remain the same, with A4 and A5 having clear segregation of nuclei based on MAT-locus with SL1 showing a much more mosaic pattern. [file elife-39813-supp12.pdf]
